# Book Reviews

**Published:** 1804-01-01

**Authors:** 


### CRITICAL ANALYSIS

OF THE

### RECENT PUBLICATIONS

ON THE

### DIFFERENT BRANCHES OF PHYSIC, SURGERY, AND MEDICAL PHILOSOPHY.

---

### *Richerand's Elements of Physiology, concluded.*

In the subject of the fourth chapter, Respiration, the reader will naturally expect a very copious addition to the labours of Haller, as many of the most interesting facts relative to this function have been discovered or elucidated since the publication of the Primæ Lineæ.

The mechanical powers by which the dilatation of the thorax is effected, are first noticed by Mr. Richerand. He very properly considers the diaphragm as almost the only agent in common dilatation of the thorax, and the muscles of the chest as only auxiliary.

"When any cause whatever renders inspiration difficult, prevents the diaphragm from descending towards the abdomen, or in any other manner impedes the motion of inspiration, the intercostal muscles not only evidently act to induce a dilatation of the thorax; but also several other auxiliary muscles, as the scaleni, subscapulares, pectorales, serrati majores, latissimi dorsi, in contracting raise the ribs, and increase the diameter of the thorax in several directions; directions; the fixed point of these muscles should then be their movable part, because the cervical spine, the clavicle, scapula, and humerus, are fixed by other powers which it would be needless to enumerate. Any person who observes the access of a convulsive asthma, or the paroxysm of a severe cough, may easily appreciate the importance and action of these auxiliary muscles."

The history of the chemical changes produced by respiration on the air inspired, and on the blood in the arterial and venous systems, together with the accruing phenomena of animal heat, is a fair subject for the proof of the Author's research and ability; we shall therefore attend to it with more minuteness. The following is the Author's description.

"Each time the thorax dilates in an adult man, from thirty to forty cubic inches of atmospheric air enter into the lungs; and when in a state of purity, composed of 73 parts of azot, 27 parts of oxygen, and $\frac{1}{100}$ or $\frac{2}{100}$ of carbonic acid.

"After the atmospheric air has remained some moments in the pulmonary structure, it is expelled by the effort of expiration; but its quantity is diminished: it is reduced to thirty-eight inches. Its composition is not the same: there are found, certainly, $\frac{73}{100}$ of azot, but the oxygen, its vital and respirable part, has suffered a great diminution; its proportion is only $\frac{14}{100}$: carbonic acid constitutes the remaining $\frac{13}{100}$, and sometimes one or two parts of hydrogen gas are found. It is likewise altered by the mixture of an aqueous vapour, which condenses in cold weather in passing out through the nostrils and mouth: it is known by the name of the humour of pulmonary exhalation. These changes, compared with those which the blood has suffered in its passage through the lungs, manifestly indicate a reciprocal action of this liquid and the oxygen of the atmosphere. The dark venous blood, slow of coagulation, and separating much serum, loaded with hydrogen and carbon, possessing only thirty degrees of heat, gives off to the oxygen of the atmosphere its hydrogen and carbon, to constitute the carbonic acid and pulmonary vapour; and as oxygen cannot enter these new combinations without disengaging a portion of caloric, which rarefies it into gas, the blood seizes this heat, now liberated with so much greater facility as it proportionally loses its hydrogen and carbon; and agreeably to the ingenious experiments of Crawford, its capacity for caloric augments in the relation of 10 : 11.5.

"The blood, in relinquishing its carbon, which, combined with oxygen, forms the carbonic acid given off in expiration, changes its dark and almost violet colour to a bright vermillion red; its consistency is increased by the dissipation of its hydrogen and aqueous parts; besides, as it absorbs a certain quantity of oxygen, it becomes frothy and lighter, its concrescibility and plasticity increase, and on coagulation less serum is separated.

"The blood, in passing through the lungs, is deprived of hydrogen and carbon, and, in becoming arterial, is loaded with oxygen and caloric. In proportion to its distance from the heart, it is deprived prived of its oxygen and its caloric, which are formed into oxyds of hydrogen and carbon; these, by a fresh addition of oxygen, when under the influence of atmospheric air in the lungs, form water and carbonic acid.

"This theory of the disoxygenation of blood, in passing through sanguiferous vessels, acquires a new degree of probability from the recent discoveries on the nature of the diamond. This body is the only pure carbon, and the substance to which chemists give this name is an oxyd of carbon, deriving its black colour from the oxygen with which it is combined. Before these experiments, it was difficult to determine the particular state of carbon that forms such a considerable part of venous blood.

"We have not yet precisely determined the respective quantities of oxygen absorbed by venous blood, and of the same oxygen employed to disengage the hydrogen and carbon in the lungs, to form the water and carbonic acid.

"Is the carbon in venous blood only combined with oxygen, or united with hydrogen, forming an hydro-carbon? It seems to me more probable that the oxygen absorbed, in uniting with the hydrogen in every part of the body, produces the water that dilutes the venous blood, renders it more fluid, and abounding in serum, than arterial blood; whilst, in uniting with carbon, it forms an oxyd that gives this blood a dark colour, constituting one of its principal characteristics. The water when in the lungs, which are true secretory organs, exhales, dissolved by the air, and forms pulmonary transpiration or exhalation; the oxyd of carbon, more completely burnt by a superaddition of oxygen, constitutes carbonic acid, which gives the air passed by expiration the property of precipitating lime-water."

A more confused and slovenly account of this most interesting proceess, we will venture to say, was never given by any one who professed to condense in one Treatise, all the elements of physiological science. Scarcely a single experiment is mentioned, on which the theory is founded; no facts, no data, no calculation; in short, nothing that indicates any research even into those sources which must be peculiarly obvious to French writers. Surely Lavoisier, Laplace, Jurine, Hassenfratz, and many others, would have furnished him with abundant matter, without the toil of research into foreign writers, of which he appears so profoundly ignorant. Neither is the meagre, scanty information which he does bestow entirely accurate. The quantity of carbonic acid stated to be produced is that of successive inspirations of the same air, not of a single inspiration, as would be inferred by the context. The following is another specimen of equal inaccuracy:

"Arterial blood becomes venous by parting with its oxygen when any cause suspends or retards its course, as proved by the following experiment of J. Hunter. He tied the carotid of a dog in two places, at about four inches distance; the blood which came out of that portion of the artery between the ligatures, when opened several hours afterwards, was coagulated and dark like that of the veins."

If the author had not previously resolved that arterial blood should become venous *by parting with its oxygen*, he would never have made this inference from Hunter's experiments, which shews only that arterial blood *remaining at rest* within the vessel assumes a venous colour.

The Section on Animal Heat is still more defective than the former, nor is its connection with respiration traced with any degree of ability or precision.

The fifth Chapter treats of secretions, and particularly of the blood, the source of all the secretions. The latter is described more chemically than physiologically, and hence many interesting particulars relative to the coagulation of blood are entirely omitted.

Of the secretions themselves, only the fat is described individually; the others are noticed in a general way along with the organs which separate them, and the secretory glands.

A trifling chapter on Nutrition succeeds, which the author appears to dispatch with great haste, that he may proceed to a long and important chapter, the subject of which is Sensation.

The order of description is, the sensations of sight, sound, smell, taste, and feeling, with their corresponding organs, the nerves, the brain, and the general functions of the nervous system. As a favourable specimen of the author's anatomical descriptions, we shall give part of that of the eye.

"The globe of the eye may be considered as a dioptric machine placed before the retina, destined to refract luminous rays, collect them into a focus, which strikes one point of this nervous membrane, exclusively adapted to receive the impression. One external, membranous, firm, and consistent covering sustains all its parts. Next to this membrane, called sclerotic, is found the choroid, a black tunic that lines the interior surface of the sclerotic, and causes the eye to be a *camera obscura*. At the anterior part of the globe the sclerotic leaves a circular opening, in which the transparent cornea is received; about the twelfth part of an inch distance from this convex segment, in the anterior aperture of the sclerotic, is found the iris, a membranous system, perforated by a round aperture (the pupil), which dilates or contracts conformably to the expansion or contraction of the iris.

"At a very small distance behind the iris, about the union of the anterior quarter of the globe of the eye with the remaining three quarters, posterior and opposite the opening of the pupil, there is a lenticular body, enclosed in a membranous capsule, invariably fixed in the place it occupies by its adherence to the membrane of the vitreous humour.

"Behind the crystalline lens the three posterior quarters of the cavity of the eye are filled with a viscous, transparent humour, enclosed in the cells of an extremely fine membrane, called a hyaloid. This vitreous humour constitutes about two-thirds of the sphere from which an anterior third might have been taken off; it is on the surface of this membrane and humour that the pulpy expansion of the optic nerve, called retina, is distributed, which adheres also as firmly to the choroid and sclerotic membranes.

"The globe of the eye being almost spherical, this difference of its diameters is very inconsiderable; the diameter from the anterior to the posterior part, is ten or eleven lines; the transverse and vertical diameters are not quite so long. In the space formed by the antero-posterior diameter that forms the visual axis, are found, in passing from before, backwards, the cornea, the aqueous humour, in the anterior chamber, the iris, and its central perforation, the aqueous humour of the posterior chamber, the chrystalline lens, surrounded by the ciliary processes, then the vitreous humour enclosed in the hyaloid membrane; and behind these transparent parts of the eye, through which the luminous rays pass in approaching the perpendicula, are, the retina, that receives the impression, then the choroid, the black coat, pigmentum nigrum, which absorbs the rays that penetrate through the fine retina; and, lastly, the sclerotic, perforated for the entrance of the optic nerve into the globe of the eye.

"The cornea enclosed in the anterior space of the sclerotic, like the glass of a watch in the frame of its outside case, is about the third of a line in thickness; it forms the segment of a smaller sphere before the eye, in addition to the anterior part of a larger sphere; behind it is the aqueous humour that fills what are called the chambers of the eye: these are distinguished into an anterior or larger space, limited by the cornea before and the iris behind; and into a posterior or smaller space, separating the crystalline lens from the iris, the posterior surface of which is covered with a black substance called *uvea*."

In the latter part of this chapter, Vicq d'Azyr and Condillac have furnished some masterly anatomical descriptions, and acute metaphysical remarks.

In the eighth chapter the phenomena of motion, and of all the combined action of the voluntary muscles, are described; and in general, in a more satisfactory and instructive way than perhaps any other subject in the whole volume. The idle chemical theory of Girtanner, to explain muscular motion, meets with more applause from our author than we expected. A short notice of Galvanism is also introduced in this part.

In the succeeding chapter on the voice, the following remarks on ventriloquism may give some information:

"There resides in the ci-devant Palais Royal, in the coffee-house of the Grotto, a man who can maintain a dialogue with such accuracy, that we should be induced to believe two persons were actually engaged in conversation at a certain distance from each other, the accent and voice of whom seem to be entirely different. I have observed that he does not inspire when speaking from the belly, but that the air passes in smaller quantity from the mouth and and nostrils than when in ordinary speech. Every time that he exerts this unusual peculiarity he suffers distension in the epigastric region; sometimes he perceives the wind rolling even lower, and cannot long continue this exertion without fatigue.

"At first I had conjectured that a great portion of the air expelled by expiration did not pass out by the mouth and nostrils, but was swallowed and carried into the stomach, reflected in some part of the digestive canal, and gave rise to a real echo; but having afterwards more attentively observed this curious phenomenon in M. Fitz-James, who represents it in its greatest perfection, I was enabled to convince myself that the name *ventriloquism* is by no means applicable, since the whole of its mechanism consists in a slow, gradual expiration, drawn in such a way that the artist either makes use of the influence exerted by volition over the muscles of the parieties of the thorax, or that he keeps the epiglottis down by the base of the tongue, the apex of which is not carried beyond the dental arches.

"He always makes a strong inspiration just before this long expiration, and thus conveys a considerable mass of air into the lungs, the exit of which he afterwards manages with such address. Therefore repletion of the stomach greatly incommodes the talent of M. Fitz-James, by preventing the diaphragm from descending sufficiently to admit of a dilatation of the thorax, in proportion to the quantity of air that the lungs should receive. By accelerating or retarding the exit of air, he can imitate different voices, and induce his auditors to a belief that the interlocutors of a dialogue, which is kept up by himself alone, are placed at different distances; and this illusion is the more complete in proportion to the perfection of his peculiar talent."

No particular remarks are suggested by the next chapter on Generation.

The work concludes with the history of ages, temperaments, varieties of the human species, death, and putrefaction. Remarks on temperament are always entertaining, as they afford much scope for elegant description, and the sallies of the imagination. Without commenting on the soundness of our author's observations, we cannot but smile at the examples or proofs which he brings in illustration. Thus, the *sanguineous* temperament is exhibited, physically, in the statues of the Antinous and Apollo; morally, in the character of Mark Anthony and Alcibiades; the *bilious* or *choleric*, in the haughty spirits of Achilles, Alexander, or Julius Cæsar; the *pituitary*, in the mild, conciliating Atticus, the common friend of irreconcilable enemies. But he does not give his proofs that Atticus had a fair skin, flaxen or sandy hair, and a slow pulse; Achilles, an excessive perfection and precosity of the liver, to the prejudice of the lymphatic and cellular systems; and Alexander, or Mahomet, a hard pulse, yellowish brown skin, and firm muscles.

The work before us will, doubtless, instruct most learners, who take the pains to study it; but we are compelled to observe, that it is is not what it professes to be, a complete outline of Physiology, according to the present state of human knowledge, being defective in those few parts in which extensive learning was required to collect widely scattered materials, and full only in those in which the Author could profit largely from the *finished* labours of his predecessors.

The Translator appears to have executed his task with general fidelity and elegance. A few errors have occurred to our notice, one of which (phosphat of lime for phosphat of iron) very unfortunately makes nonsense of an explanation of the red colour of the blood; and, for the convenience of the English reader, the degrees of Reaumur's thermometer ought universally to have been expressed by their equivalents on the English scale.

---

*Observations on the anti-phthisical Properties of the Lichen Islandicus, or Iceland Moss; comprehending explicit Directions for the making and using such Preparations of the Herb and Auxillaries, which experience has proved best adapted to the Cure of the different Species of pulmonary Consumptions, of Great Britain; by* Rich. Reece. 8vo. pp. 32. 1803.

The principal object of this short pamphlet appears to be, to suggest what the author considers as improvements upon Dr. Regnault's mode of employing the lichen. These are founded on a division of the properties of the plant into the bitter and the mucilaginous parts, and combining them, according to the indications, with other auxillaries.

"The Lichen Islandicus (says Mr. R.) possesses considerable medicinal and dietetic properties; the latter residing in a strong mucilage, which affords a regimen well adapted to support the debilitated frame of phthisical patients; and the former in a bitter, which, in proper doses, is evidently of an anodyne nature, which powerfully allays cough; and, unlike opium, at the same time facilitates expectoration, abates hectic fever, quiets the system, without constipating the bowels. It is likewise tonic, which strengthens the organs of digestion; and different from any other of that class, without increasing the action of the heart and arteries—the union of these properties unquestionably affords a most valuable remedy in the treatment of pulmonary consumption.

"The bitter portion of this herb, (which must be considered the principal agent in the relief of the phthisical symptoms) is readily imparted to boiling water by infusion; but by the long boiling necessary to extract its mucilage, this quality is nearly destroyed.—Quarin, sensible of this circumstance, directs the herb to be boiled in water only half an hour, which extracts but a small portion of the mucilage, and contains its medicinal virtues, unimpaired; and Hartmann recommends two drachms of the herb to be boiled in a pint of milk a short time, which is ordered to be drank off in a morning; and this form has been most followed by the physicians

 in in London; but with patients affected with dyspeptic symptoms, this medium often proves too heavy."

Our observations do not confirm these assertions; we do not find the bitter quality of the herb destroyed by the boiling necessary to extract its mucilage; if the boiling be properly conducted, which should be only digesting in a boiling heat, the bitter will not be diminished. The practice imputed to the physicians in London, of recommending a slight boiling of the plant in milk, is contradicted by our information, and we should be surprised to find any such practice generally prevalent in the metropolis. If, however, medical men should be desirous of combining the virtues of the lichen with a milk diet, they can be at no loss to reconcile them to dyspeptic habits.

The author agrees with Dr. R. p. 6, in recommending a full and free use of the plant, both as diet and medicine; but he prefers a flour, or, as he terms it, a *farina*, made from the herb, which he thinks may be converted into the suitable preparations more easily than the plant itself. This indeed is the practice of the Icelanders, who make it a principal article in their soups or broths, and often make bread of it; but their mode of grinding it, or any other that could be employed in private families, is too troublesome for general use. We have therefore been in the habit of recommending the digestion in a boiling heat for two or three hours; and when the liquor has been strained, as much of the herb is to be rubbed thro' the sieve as can be made to pass, and mixed with the liquor. By this process all the virtues of the plant will be obtained, without the trouble of grinding it; the danger of sophistication, to which the temptation is so great, and the detection so difficult when the meal is purchased ready ground, will also be avoided. Though Mr. R. may prepare it faithfully, others may adulterate; and there is no exclusive privilege to guard us against frauds.

We are happy to find that Dr. Regnault has succeeded in drawing the public attention so generally to this subject, and that he has been called upon for a second edition of his Essay. In consequence of such request, we understand he has made considerable additions and improvements both in the pathology of consumption and the manner of treating its different stages; and that the work will make its appearance in the course of a few days.

---

*The London Practice of Midwifery, or a Manual for Students.* London, 1803.

THIS work professes to be a System of Midwifery, founded upon the London practice, but we have authority to say that it is only a surreptitious copy of the notes taken at the Lectures of an individual Teacher of Midwifery in London. We wish that we could even recommend it as such; but the whole work bears evident marks of haste and inaccuracy. The very names of writers are falsely spelt, as Peuso for Puzos, Bourdelois for Baudeloque, &c.

Words

Words are given as Ambrose Paré's which are not to be found in his works; and Haller is made to vouch for opinions, which he never entertained. Dr. Johnson is quoted as the author of experiments which he never made or thought of; experiments of the late Mr. Hunter are related in the most inaccurate manner; and experiments of Spalanzani are positively attributed to Mr. Hunter. Some passages may be found, which are absolute nonsense, and such as no Teacher could ever have delivered; for example, "The menstrual discharge from the uterus serves as a nidus, in which the ovum is nourished."

We have noticed these among an infinite number of errors in the physiological part.

Innumerable mistakes are to be found in the practical part of this work, which are more important, because they are capable of doing more mischief, and of misleading young practitioners. In the rules for the application of the forceps, the *sides* are mistaken for the edges of the instrument, so that if it was applied by the rules laid down infinite harm may be done.

On the whole we must repeat, that the book is a very bad compilation from the notes hastily and very incorrectly taken by a young student, at the lectures of an individual teacher, and is too incompleat to serve as a practical guide to those for whom manuals of this kind are intended.

